# TCR/pMHC Optimized Protein crystallization Screen

**DOI:** 10.1016/j.jim.2012.06.007

**Published:** 2012-08-31

**Authors:** Anna M. Bulek, Florian Madura, Anna Fuller, Christopher J. Holland, Andrea J.A. Schauenburg, Andrew K. Sewell, Pierre J. Rizkallah, David K. Cole

**Affiliations:** Institute of Infection and Immunity, Cardiff University School of Medicine, Heath Park, Cardiff, CF14 4XN, UK

**Keywords:** Crystal structure, Peptide-major histocompatibility complex (pMHC), T cell receptor (TCR), Crystallization, X-ray diffraction, High throughput crystallization screen

## Abstract

The interaction between the clonotypic αβ T cell receptor (TCR), expressed on the T cell surface, and peptide-major histocompatibility complex (pMHC) molecules, expressed on the target cell surface, governs T cell mediated autoimmunity and immunity against pathogens and cancer. Structural investigations of this interaction have been limited because of the challenges inherent in the production of good quality TCR/pMHC protein crystals. Here, we report the development of an ‘intelligently designed’ crystallization screen that reproducibly generates high quality TCR/pMHC complex crystals suitable for X-ray crystallographic studies, thereby reducing protein consumption. Over the last 2 years, we have implemented this screen to produce 32 T cell related protein structures at high resolution, substantially contributing to the current immune protein database. Protein crystallography, used to study this interaction, has already extended our understanding of the molecular rules that govern T cell immunity. Subsequently, these data may help to guide the intelligent design of T cell based therapies that target human diseases, underlining the importance of developing optimized approaches for crystallizing novel TCR/pMHC complexes.

## 1 Introduction

T cells play an important role in the protection against pathogens and cancer and have been shown to cause/contribute towards many autoimmune diseases (Wong and Pamer, 2003; Rudolph et al., 2006; Bulek et al., 2012). The T cell receptor (TCR) recognizes foreign and self protein fragments bound to the self-major histocompatibility complex (pMHC) (Garboczi et al., 1996). The first structure of a murine TCR (2C) with MHC class I H2-K^b^ in association with dEV8 peptide was published in 1996 (Garcia et al., 1996). This was shortly followed by the structure of a human TCR (A6) in complex with HLA-A*0201-Tax (peptide derived from human T cell lymphotropic virus type 1_11–19_) (Garboczi et al., 1996). These structures provided the first insight into T cell antigen recognition and revealed a number of important features of the interface between the TCR and pMHC. Ten years later, only 10 unique human TCR/pMHC complexes had been solved, as reviewed by Rudolph et al. (2006). In recent years, this number has increased to ~ 25 human TCR/pMHC complexes, but progress has still been relatively slow compared with the number of antibody structures, or unligated pMHC structures that have been reported. This lack of structural information regarding human TCR/pMHC complexes has compromised the determination of a comprehensive and accepted set of rules that govern T cell antigen recognition and a number of conflicting theories still dominate the field (Bridgeman et al., 2012).

Difficulties in generating sufficient quantities of soluble TCR and pMHC protein, and in producing high quality TCR/pMHC complex crystals, may explain the low number of these structures. Additionally, TCRs bind to pMHCs with relatively weak affinity (K_D_ = 0.1–300 μM (Cole et al., 2007; Bridgeman et al., 2012)), which may further impede their ability to form stable complexes for crystallization. A number of approaches have been proposed for the production of stable, soluble recombinant TCRs, including modification of the expression vectors and optimization of culture conditions. To date, soluble TCRs have been generated using various eukaryotic expression systems such as: *Drosophila melanogaster* (Garcia et al., 1996), myeloma cells (Wang et al., 1998), Chinese hamster ovary cells (Reiser et al., 2000) and *Spodoptera frugiperda* cells (Hahn et al., 2005). However, prokaryotic expression as inclusion bodies using *Escherichia coli* strains, followed by artificial refolding, remains the most popular and robust system because it produces high yields of homogenous protein (Cole et al., 2007, 2008, 2009). Additionally, four different TCR cloning methods have been designed to improve soluble TCR stability including: (1) expression of the variable domains only in a form of a single chain Fv fragment (scFv) (Housset et al., 1997); (2) expression of TCR α and β chains carrying c-Jun (α) and c-Fos (β) leucine-zipper heterodimerization motifs at their carboxyl termini (Garcia et al., 1996); (3) introduction of a carboxy-terminal flanking sequence to the full length V and C ectodomains to promote the formation of an interchain disulphide bridge (Stewart-Jones et al., 2003); and, (4) introduction of a non-native disulphide bond into the interface between the TCR constant domains (Boulter et al., 2003). The ‘Boulter-disulphide’ method has been the preferred choice in our laboratory.

Once expressed and purified, the last challenge is to generate TCR/pMHC complex protein crystals capable of high resolution X-ray diffraction. In order to achieve this, a number of commercial screens, not tailored specifically for T cell associated proteins, have been used by different laboratories with some success (evidenced by the modest number of TCR/pMHC complexes published). Here we report the development of a new crystallization screen specifically designed for the production of high quality TCR, pMHC and TCR/pMHC complex crystals suitable for crystallographic studies. A wide selection of TCRs, pMHCs and TCR/pMHC complexes, implicated in variety of diseases, were used to test the efficacy of our screen. Using this novel approach, we have been able to generate 32 crystal structures comprising: 21 TCR/pMHC complexes, 3 TCRs and 8 pMHCs, over the last 2 years. These structures have already enabled a better understanding of T cell antigen recognition of viral (Miles et al., 2010), autoimmune (Bulek et al., 2012) and cancer (Cole et al., 2009) epitopes, as well as a number of so far unpublished observations. Thus, our *T*CR/pMHC *O*ptimized *P*rotein crystallization *S*creen (TOPS) will allow us, and others, to investigate many important questions regarding the molecular basis of T cell mediated immunity.

## 2 Materials and methods

### 2.1 Cloning and expression

The TCR α and TCR β chains, as well as the MHC class I α chain and β2m sequences, were cloned into the pGMT7 expression vector under the control of the T7 promoter using BamH1 and EcoR1 restriction sites as described previously (Garboczi et al., 1992, 1996; Boulter et al., 2003). Sequences were confirmed by automated DNA sequencing.

The TCR α and β chains, as well as HLA A*0201 α chain and β2m were expressed separately, without post-translational modification, as insoluble inclusion bodies (IBs) in competent Rosetta DE3 *E. coli* cells as described previously (Garboczi et al., 1992, 1996; Boulter et al., 2003).

### 2.2 Refolding and purification

TCR refolding was performed as previously reported (Miles et al., 2010). Briefly, for a 1 L TCR refold, 30 mg TCR α-chain IBs was incubated at 37 °C for 15 min with 10 mM DTT and added to cold refold buffer (50 mM TRIS, pH 8.1, 2 mM EDTA, 2.5 M urea, 6 mM cysteamine hydrochloride, and 4 mM cystamine). After 15 min, 30 mg TCR β-chain IBs, incubated at 37 °C for 15 min with 10 mM DTT, was added to the same refold. For a 1 L pMHC class I refold, 30 mg HLA A*0201 α-chain was mixed with 30 mg β2m and 4 mg peptide at 37 °C for 15 min with 10 mM DTT. This mixture was then added to cold refold buffer (50 mM TRIS, pH 8, 2 mM EDTA, 400 mM l-arginine, 6 mM cysteamine hydrochloride, and 4 mM cystamine). Refolds were mixed at 4 °C for > 1 h. Dialysis was performed against 10 mM TRIS, pH 8.1, until the conductivity of the refolds was less than two millisiemens per centimeter. The refolds were then filtered, ready for purification steps.

Refolded proteins were purified initially by ion exchange using a Poros50HQ™ column (GE Healthcare, Buckinghamshire, U.K.) and finally gel filtered into a crystallization buffer (10 mM TRIS pH 8.1 and 10 mM NaCl) using a Superdex200HR™ column (GE Healthcare, Buckinghamshire, U.K.). Protein quality, either under non-reducing or reducing conditions, was analyzed by Coomassie-stained SDS-PAGE.

### 2.3 Protein crystallization

Crystals were grown at 18 °C by vapor diffusion via the sitting drop technique. All crystallization screening and optimization experiments were completed with an Art-Robbins Phoenix dispensing robot (Alpha Biotech Ltd, U.K.). 200 nL of 10–20 mg/ml TCR, pMHC, or TCR and pMHC complex mixed at a 1:1 molar ratio, was added to 200 nL of reservoir solution. Intelli-plates were then sealed and incubated in a crystallization incubator (18 °C) (Molecular Dimensions) and analyzed for crystal formation. Crystals selected for further analysis were cryoprotected with 25% ethylene glycol and then flash cooled in liquid nitrogen in Litho loops (Molecular Dimensions).

### 2.4 Structure determination and refinement

Diffraction data was collected at a number of different beamlines at the Diamond Light Source, Oxford, using a Pilatus 2M, or a QADSC, detector. Using a rotation method, 400 frames were recorded each covering 0.5° of rotation. Reflection intensities were estimated with the XIA2 package (Winter, 2010) and the data were scaled, reduced and analyzed with SCALA and the CCP4 package (Collaborative Computational Project, N, 1994). The TCR, pMHC, or TCR/pMHC complex structures were solved with molecular replacement using PHASER (McCoy et al., 2005), or AMORE (Trapani and Navaza, 2008). The model sequences were adjusted with COOT (Emsley and Cowtan, 2004) and the models refined with REFMAC5.

## 3 Results

### 3.1 Design of a *T*CR/pMHC *O*ptimized *P*rotein crystallization *S*creen (TOPS)

**Fig. 1:**
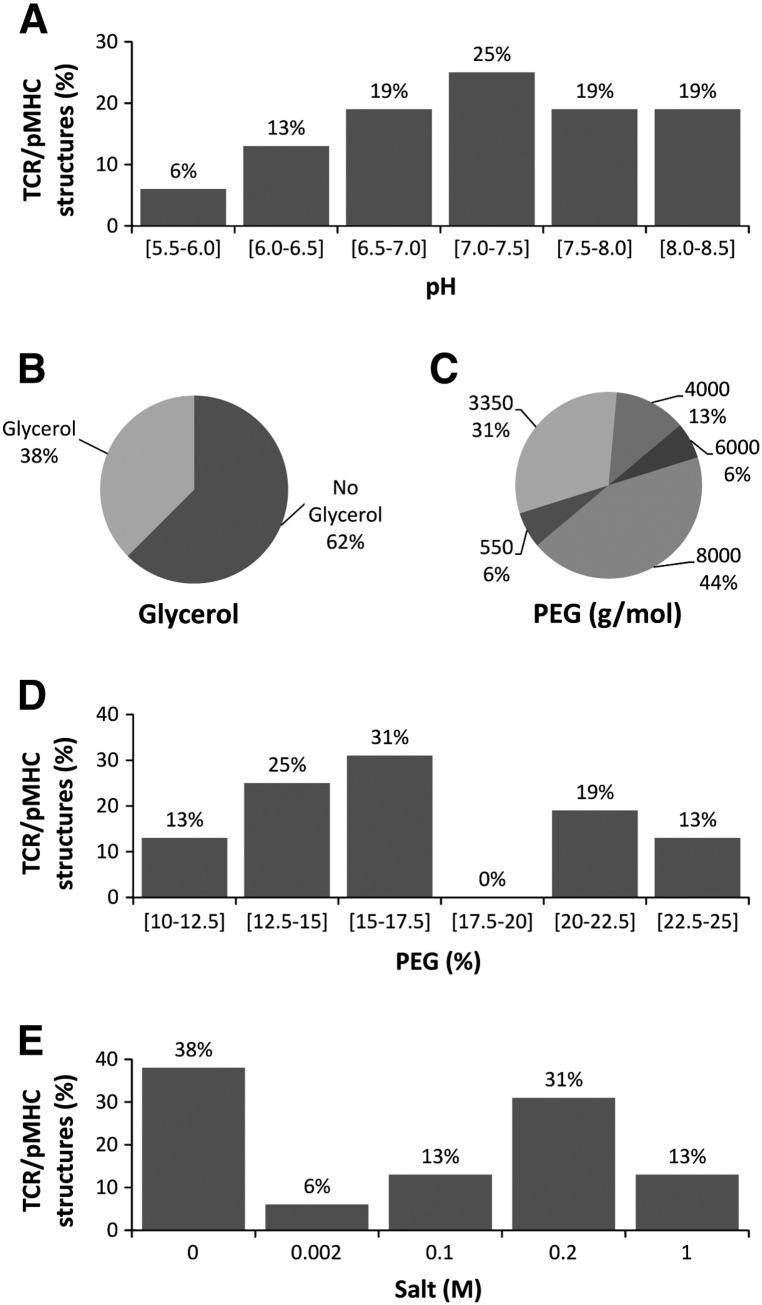
Analysis of the crystallization conditions of 16 previously published TCR/pMHC complexes. (A) The pH of the conditions lay between pH 5.6–8.5, with a preference for the higher end of this pH range. (B) Glycerol was used as a cryo-protectant in 38% of cases. (C) The molecular weight of the PEG varied from 550 g/mol to 8000 g/mol (PEG 3350, 4000 and 8000 being the most successful). (D) The best PEG concentration was between 15%–17.5%, with a PEG concentration range from 10%–25%. (E) Various salts, with concentrations from 0–1 M, were used, with a preference for 0.2 M.

TCR/pMHC complex structures have previously been solved by a number of different groups using individually determined crystallization conditions. In order to combine these data to generate a comprehensive *T*CR/pMHC *O*ptimized *P*rotein crystallization *S*creen (TOPS), we investigated the crystallization conditions of 16 previously published TCR/pMHC complexes (Garboczi et al., 1996; Garcia et al., 1996; Ding et al., 1998, 1999; Hennecke et al., 2000; Reiser et al., 2000, 2003; Hennecke and Wiley, 2002; Kjer-Nielsen et al., 2003; Stewart-Jones et al., 2003; Chen et al., 2005; Li et al., 2005; Maynard et al., 2005; Tynan et al., 2005, 2007; Sami et al., 2007; Cole et al., 2009) (Fig. 1). Although there was a substantial variation in the crystallization conditions identified for different TCR/pMHC complexes, we noticed certain trends. The pH lay between 5.6–8.5 in all cases, with the TCR/pMHC complexes tending to crystallize at the higher end of this pH range (Fig. 1A); with 25%, 19% and 19% of complexes crystallizing in the pH range of 7.0–7.5, 7.5–8.0 and 8.0–8.5, respectively. Six conditions (38%) contained glycerol as cryoprotectant (Fig. 1B). All conditions contained PEG (polyethylene glycol), although the weight (550–8000 g/mol) and percentage (10–25%) were very variable. The best PEG concentration, representing 31% of the previous structures reported, was between 15%–17.5%. Molecular weight PEG 3350, 4000 and 8000 were most successful (Fig. 1C and D) evidenced by 31%, 13% and 44% of all structures being obtained with each additive, respectively. Another common component of successful conditions were various salts, with concentrations ranging from 0–1 M, the absence of salt (38%) and 0.2 M (31%) being most popular (Fig. 1E).

Based on these findings we developed a crystallization screen for TCR/pMHC complexes (Tables 1A and 1B). Our screen consisted of two 48 well PEG/pH screens. Each PEG/pH screen consisted of four buffer systems (C_2_H_6_AsO_2_Na, MES, HEPES and TRIS) at a concentration of 0.1 M in combination with PEG 4000, or PEG 8000 at 15, 20 and 25%. These buffers allowed scanning the pH range from 6.0–8.5. 15% glycerol was added to the first subscreen (Table 1A), whereas 0.2 M ammonium sulfate was added to the second subscreen (Table 1B).

In some cases, TOPS generated several crystal hits that were of lower quality, i.e. the crystals were very small, contained cracks or impurities, or did not diffract to high resolution. In these cases, we extended the conditions that yielded crystals to generate a number of other fine screens that proved useful for specific TCR/pMHC complexes. TOPS1 (Supplementary Table 1) was designed by extending the lower range of pH with C_2_H_6_AsO_2_Na pH 5.0 and 5.5 of the A07 condition of the TOPS screen. In addition, PEG 3350 was compared versus PEG 4000 in this screen. TOPS2 (Supplementary Table 2) was designed by extending the lower range of PEG concentration (10, 12.5, 15, 17.5, 20 and 22.5%) of the second subscreen of the TOPS screen. In addition, one of the buffer systems (C_2_H_6_AsO_2_Na pH 6.0) was replaced by a non-buffered condition and supplemented by another precipitant (0.2 M sodium sulfate) as some good hits were obtained using a commercially available screen (PACTPremier, condition E08; 0.2 M sodium sulfate and 20% PEG 4000). TOPS3 (Supplementary Table 3) was designed by reducing the range of pH (from 6.5–7.5) and increasing the number of buffer system (MES pH 6.5, BIS TRIS propane pH 7.0 and TRIS pH 7.0) as well as the range of glycerol concentrations (0, 4.4, 8.7 and 17.4%). PEG 4000 was the PEG of choice in this screen. The only difference between TOPS3 and TOPS4 (Supplementary Table 4) was that TOPS4 contained 0.2 M ammonium sulfate. These screens generated 5 TCR/pMHC complexes as detailed in Table 2.

### 3.2 Optimal conditions for the generation of TCR/pMHC complex crystals

High-throughput crystallization trials were performed using 3 commercially available screens (PACT Premier, JBScreen and JCSG-plus (Molecular Dimensions Ltd, Suffolk, U.K.)) and/or 5 different “homemade” screens (TOPS, TOPS1, TOPS2, TOPS3 and TOPS4) (Tables 1A and 1B, Supplementary Tables 1–4), the last four screens being derivatives of the TOPS screen. Crystallization conditions were successfully identified for 25 TCR/pMHC complexes, 14 of which were derivatives from a common parent complex. Among these 25 unique complexes, 21 were obtained from the TOPS screen or TOPS screen-derived conditions while only 3 were obtained from the PACT Premier screen and 1 from the JBScreen. No complexes were obtained from the JCSG-plus screen. Thus, TCR/pMHC structures that crystallized in TOPS screen represented more than 80% of the total number of complexes solved (Table 2).

Although the TOPS screen was designed for TCR/pMHC complexes, a selection of uncomplexed TCR and pMHC proteins were generated based on our ongoing research interests, to test the efficacy of TOPS. This approach directly resulted in structures of 3 uncomplexed TCR and 8 pMHC proteins. The total number of 25 complexes and 53 datasets (we often collected several datasets from different conditions for a particular complex) allowed us to perform an analysis in order to define the most optimal conditions for growing crystals of TCR/pMHC complexes.

**Fig. 2:**
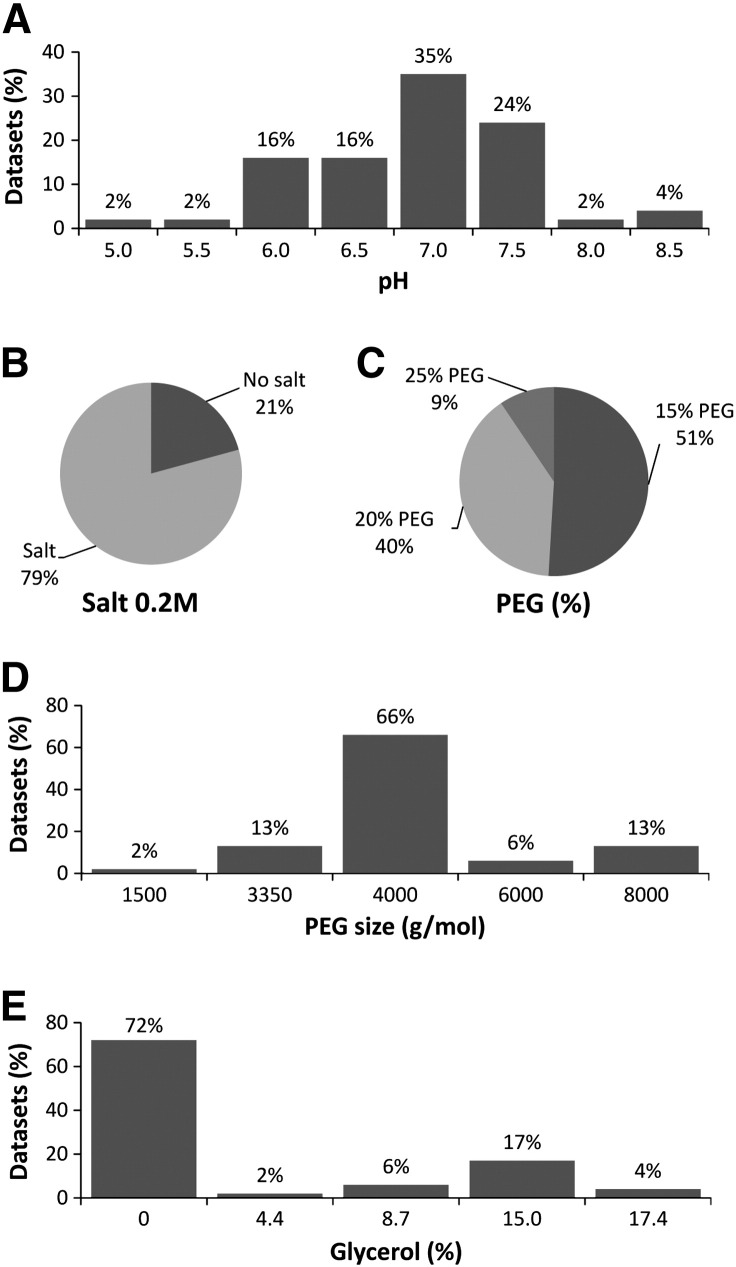
Analysis of crystallization conditions obtained from 25 TCR/pMHC complexes. (A) The pH was within a range of 5.0–8.5, with 91% of the datasets obtained around a neutral pH from 6.0–7.5. (B) The presence of 0.2 M salt was required in 79% of the conditions. (C) The best PEG concentrations were 15% and 20%, representing 91% of the datasets. (D) The most popular PEG molecular weight was around 4000 g/mol, representing 79% of the datasets (13% PEG 3350 and 66% PEG 4000). (E) The absence of glycerol was dominant (72%), but the best concentration of glycerol when this component was required was 15%.

Crystallization conditions are presented in Fig. 2. In all cases, the pH was within a range of 5.0–8.5. However, the great majority of crystals (90%) were obtained around a neutral pH of 6.0–7.5, and more than a third (35%) at pH 7.0 (Fig. 2A). The presence of salt, a precipitating agent, at 0.2 M was required as 79% of crystals successfully grew in such conditions (Fig. 2B). The best PEG concentrations, another precipitating agent, were 15% and 20%, resulting in 51% and 40% of the datasets, respectively. In contrast, higher precipitant concentrations produced only 9% of the datasets (Fig. 2C). The most popular PEG size was around 4000 g/mol with 79% of datasets obtained in this condition (13% PEG 3350 and 66% PEG 4000). PEG at smaller molecular weight only generated 2% of the datasets, whereas PEG at higher molecular weight generated 19% of the datasets (6% and 13% of PEG 6000 and 8000 respectively) (Fig. 2D). Although glycerol was a good cryoprotecting agent, the absence of this component was essential in 72% of the cases. However, when the presence of glycerol was required, 15% appeared to be the best concentration (Fig. 2E).

**Fig. 3:**
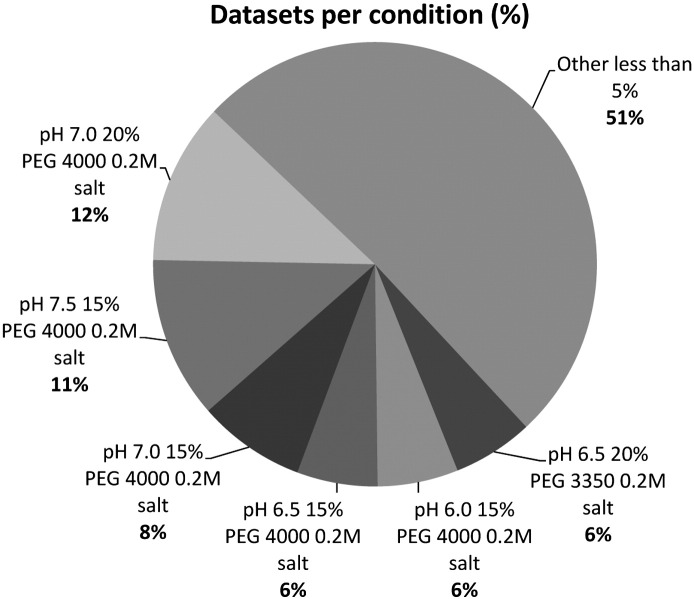
Analysis of the frequency of appearance of a particular condition. The conditions producing less than 5% of the datasets were combined together. The 53 datasets were obtained in a total of 29 different conditions. Among these 29 conditions, 6 conditions (pH 6.5 20% PEG 3350 0.2 M salt, pH 6.0 15% PEG 4000 0.2 M salt, pH 6.5 15% PEG 4000 0.2 M salt, pH 7.0 15% PEG 4000 0.2 M salt, pH 7.5 15% PEG 4000 0.2 M salt and pH 7.0 20% PEG 4000 0.2 M salt) produced nearly half of all datasets.

**Fig. 4:**
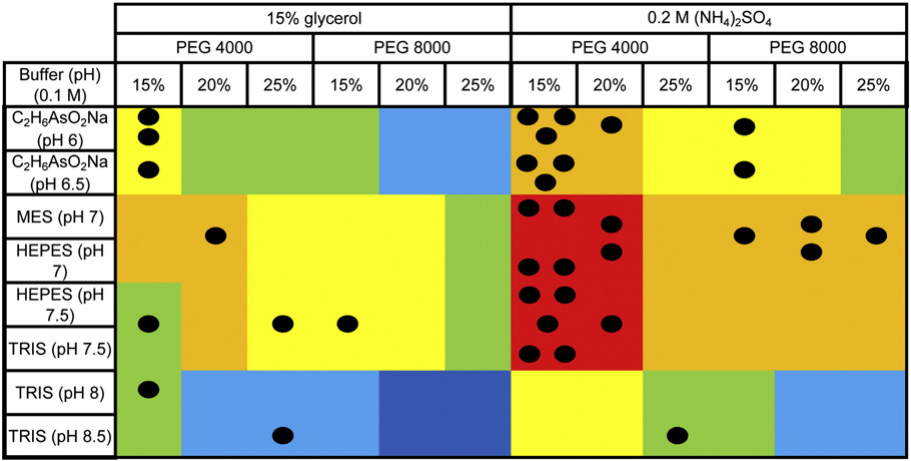
Representation of the expected TOPS efficiency correlated to the datasets obtained with that screen. Based on the analysis presented in Fig. 2, we designed a scoring system related to the percentage of datasets obtained per condition of pH, PEG concentration, PEG size and the presence of salt or glycerol. This scoring system allows us to generate a score for each condition of TOPS based on the number of TCR/pMHC complexes crystallized in each condition. We represented scores < 2.5 in dark blue, 2.5–5 in light blue, 5–7.5 in green, 7.5–10 in yellow, 10–12.5 in orange and > 12.5 in red. Each dark circle represents a successful dataset. The vast majority of TCR/pMHC complexes crystallized in the conditions with a high score, but a number of complexes crystallized in the low range of this scoring system.

Although this analysis suggested the optimal conditions for obtaining TCR/pMHC complexes, it was performed by taking each variable independently. In order to verify if a given condition was more representative than the others, the frequency of appearance of each particular condition was calculated (Fig. 3). The conditions producing less than 5% of the datasets were combined together. This combined fraction of 23 different conditions correlated to 51% of all datasets. The remaining 6 conditions (pH 6.5 20% PEG 3350 0.2 M salt, pH 6.0 15% PEG 4000 0.2 M salt, pH 6.5 15% PEG 4000 0.2 M salt, pH 7.0 15% PEG 4000 0.2 M salt, pH 7.5 15% PEG 4000 0.2 M salt and pH 7.0 20% PEG 4000 0.2 M salt), surprisingly, produced nearly half of all datasets (Fig. 3). This analysis completely correlated with the previous independent analysis with a pH range from 6.0–7.5, a required presence of 0.2 M salt, a preferred PEG size around 4000 g/mol and PEG concentrations of 15% and 20%. Based on these analyses, it could be possible to significantly restrain the crystallization conditions for TCR/pMHC complexes. However, further analysis revealed that, although the vast majority of TCR/pMHC complexes crystallized within the remit of these conditions, a number of structures crystallized in conditions outside of this range (Fig. 4). Thus, although it could be tempting to limit the number of conditions in a protein crystal screen to improve efficiency and reduce protein consumption, broader screens are required to ensure that crystallization conditions are not missed for important proteins.

## 4 Discussion

The ability of T cells to respond to antigen depends on the productive interaction between the TCR and pMHC. The crystal structures of a number of TCR/pMHC complexes have been solved and show that the TCR has a relatively conserved mode of binding to pMHC in which the TCR lines up approximately diagonally to the MHC peptide binding groove, with the TCR α chain contacting the MHC α2 domain and the TCR β chain contacting the MHC α1 domain. The antigen specific portion of the TCR/pMHC interaction occurs between the pMHC surface and the TCR complementarity determining region loops (CDR-loops) (Rudolph et al., 2006). These CDR-loops serve different roles during TCR binding to pMHC: the variable (V)-gene encoded CDR2-loops contact mainly the conserved helical region of the MHC surface, the V-gene encoded CDR1-loops can contact both the MHC and the peptide and the more variable somatically rearranged CDR3-loops contact mainly the antigenic peptide. Although the general features of TCR/pMHC binding have been defined, there remains a number of conflicting models that describe the structural basis of T cell MHC-restriction, cross-reactivity, autoimmunity and alloreactivity. Furthermore, each previous TCR/pMHC complex has been governed by a unique set of contacts that enable T cell antigen recognition. Thus, there is still a pressing need to increase the number of TCR/pMHC complex structures in the literature in order to: (1) determine an accepted set of rules that describe the generalities of T cell specificity, and (2) understand the unique features of individual TCR/pMHC interactions that allow T cells to target different disease epitopes.

The study of TCR/pMHC complexes has been limited by the challenges in expression, purification and successful crystallization of these soluble proteins. Here, we report a new systematic and directed approach for the design of a *T*CR/pMHC *O*ptimized *P*rotein crystallization *S*creen (TOPS) that has proved to be useful for the crystallization of this family of immuno-proteins. With this novel crystallization screen, we have successfully generated the majority of our current portfolio of structures that includes 21 TCR/pMHC complexes (13 derived from a common parent complex), 3 TCRs and 8 pMHCs. We found that TCR/pMHC complex crystals most commonly formed at a neutral pH, with 15%–20% of PEG 4000 and 0.2 M ammonium sulfate. In addition, results from our crystallization trials indicated that it may be possible to significantly restrain the crystallization conditions of TCR/pMHC complexes to around 6 different conditions (rather than 96). Although this could substantially reduce the quantity of protein required for the successful generation of TCR/pMHC complex crystals capable of diffracting to high resolution, our analyses revealed that a limited screen could exclude some important crystallization conditions for some proteins. Thus, our TOPS screen remains optimal for the crystallization of TCR/pMHC complexes.

In conclusion, we hope that TOPS will greatly contribute to a better understanding of molecular basis for T cell recognition of self, foreign (microbial/viral/parasitic) and autoimmune antigens by providing an improved method for generating TCR/pMHC complex protein crystals capable of high quality X-ray diffraction. Furthermore, we expect that TOPS will be useful for the determination of TCR structures in complex with classical and non-classical MHC ligands that are less well characterized, including: pMHC class II, MR1, CD1c and HLA-E. Structural information, detailing the precise atomic contacts that mediate T cell immunity, can provide clear insights into various immune dysfunctions and could accelerate the rational design of T cell based therapies and vaccines.

## Author contributions

D.K.C., C.J.H., P.J.R., A.J.A.S., A.F., A.M.B and F.M., performed experiments, analyzed data and critiqued the manuscript. D.K.C., and P.J.R., conceived and directed the project. F.M., A.M.B., D.K.C., A.K.S., and P.J.R., wrote the manuscript.

## Competing financial interests

The authors declare no competing financial interests.

## The use of animals

No animals were used in this study.

## Human samples

All human samples were used in accordance with UK guidelines.

## Figures and Tables

**Table 1A:** TOPS screen conditions 1–48.

Buffer (pH) (0.1 M)	PEG 4000			PEG 8000		
	15%	20%	25%	15%	20%	25%
C_2_H_6_AsO_2_Na (pH 6)	A1	A2	A3	A4	A5	A6
C_2_H_6_AsO_2_Na (pH 6.5)	B1	B2	B3	B4	B5	B6
MES (pH 7)	C1	C2	C3	C4	C5	C6
HEPES (pH 7)	D1	D2	D3	D4	D5	D6
HEPES (pH 7.5)	E1	E2	E3	E4	E5	E6
TRIS (pH 7.5)	F1	F2	F3	F4	F5	F6
TRIS (pH 8)	G1	G2	G3	G4	G5	G6
TRIS (pH 8.5)	H1	H2	H3	H4	H5	H6

All conditions contained 15% glycerol.

**Table 1B:** TOPS screen conditions 49–96.

Buffer (pH) (0.1 M)	PEG 4000			PEG 8000		
	15%	20%	25%	15%	20%	25%
C_2_H_6_AsO_2_Na (pH 6)	A7	A8	A9	A10	A11	A12
C_2_H_6_AsO_2_Na (pH 6.5)	B7	B8	B9	B10	B11	B12
MES (pH7)	C7	C8	C9	C10	C11	C12
HEPES (pH 7)	D7	D8	D9	D10	D11	D12
HEPES (pH 7.5)	E7	E8	E9	E10	E11	E12
TRIS (pH 7.5)	F7	F8	F9	F10	F11	F12
TRIS (pH 8)	G7	G8	G9	G10	G11	G12
TRIS (pH 8.5)	H7	H8	H9	H10	H11	H12

All conditions contained 0.2 M (NH_4_)_2_SO_4_.

**Table 2:** Successfully crystallized TCR/pMHC complexes.

TCR/pMHC complex	Screen	Resolution (Å)	pH	PEG (%)	PEG	Glycerol (%)	Salt (M)
1E6/A2-ALW	TOPS	2.6	6.0	15	4000	15	0
1E6/A2-AQW	PACT	3.0	6.5	20	4000	0	0.2
1E6/A2-RQW	PACT	2.3	6.0	20	6000	0	0.2
1E6/A2-YQF	PACT	2.1	7.0	20	6000	0	0.2
1E6/A2-WQY	TOPS	1.9	7.0	25	8000	0	0.2
1E6/A2-KLP	TOPS	2.9	6.5	15	8000	0	0.2
1E6/A2-YLG	TOPS	2.5	6.5	15	4000	0	0.2
1E6/A2-MVW	TOPS	2.0	7.5	15	4000	0	0.2
1E6/A2-RQF(I)	TOPS	1.9	7.5	15	4000	0	0.2
1E6/A2-RQF(A)	TOPS	1.9	7.0	15	4000	0	0.2
GP100/A2-YLE	JBS	2.0	7.0	20	4000	0	0.2
ILA/A2-ILA	TOPS	2.6	7.0	20	8000	0	0.2
AS01/A2-GLC	TOPS	2.6	6.0	20	4000	0	0.2
α24β17/A2-ELA	TOPS4	2.4	7.0	20	4000	0	0.2
α24β17/A2-AAG	TOPS	2.8	7.0	20	4000	15	0
α24β17/A2-ELA-4A	TOPS3	2.7	7.0	15	4000	17.4	0
α24β17/A2-ELA-7A	TOPS4	2.6	7.0	20	4000	17.4	0.2
α24β17/A2-EAA	TOPS4	2.1	7.5	15	4000	8.7	0.2
MEL5/A2-EAA	TOPS	3.0	6.5	15	4000	15	0
MEL5/A2-AAG	TOPS	3.2	7.5	25	4000	15	0
P1/A2-CLG	TOPS	2.6	6.0	15	4000	15	0
SB7/A2-FLY	TOPS	2.6	6.5	15	4000	0	0.2
868/A2-SLY	TOPS	2.9	6.0	15	4000	0	0.2
868/A2-6I	TOPS	2.9	6.0	15	4000	0	0.2
868/A2-3F6I8V	TOPS1	2.8	5.5	15	4000	0	0.2
