# Supplementary material for: TCR/pMHC Optimized Protein crystallization Screen
Source: J Immunol Methods. 2012 Aug 31;382(1-2):203–10. doi: 10.1016/j.jim.2012.06.007 (PMC3404460; doi:10.1016/j.jim.2012.06.007)
Supplement: Supplementary file 1 — Supplementary materials. [file mmc1.doc]

1. **Supplementary tables**

**Table 1A: TOPS1 screen conditions 1-30**

| Buffer (pH) (0.1 M) |  | | | | | | PEG (g/mol) (15%) |
| --- | --- | --- | --- | --- | --- | --- | --- |
| C_2_H_6_AsO_2_Na (pH 5.5) | A1 | A2 | A3 | A4 | A5 | A6 | 3350 |
| C_2_H_6_AsO_2_Na (pH 6) | B1 | B2 | B3 | B4 | B5 | B6 | 3350 |
| C_2_H_6_AsO_2_Na (pH 5) | C1 | C2 | C3 | C4 | C5 | C6 | 4000 |
| C_2_H_6_AsO_2_Na (pH 5.5) | D1 | D2 | D3 | D4 | D5 | D6 | 4000 |
| C_2_H_6_AsO_2_Na (pH 6) | E1 | E2 | E3 | E4 | E5 | E6 | 4000 |

All conditions contained 0.2 M ([N](http://en.wikipedia.org/wiki/Nitrogen)[H](http://en.wikipedia.org/wiki/Hydrogen)_4_)_2_[S](http://en.wikipedia.org/wiki/Sulfur)[O](http://en.wikipedia.org/wiki/Oxygen)_4_

**Table 1B: TOPS1 screen conditions 31-60**

| Buffer (pH) (0.1 M) |  | | | | | | PEG (g/mol) (15%) |
| --- | --- | --- | --- | --- | --- | --- | --- |
| C_2_H_6_AsO_2_Na (pH 5.5) | A1 | A2 | A3 | A4 | A5 | A6 | 3350 |
| C_2_H_6_AsO_2_Na (pH 6) | B1 | B2 | B3 | B4 | B5 | B6 | 3350 |
| C_2_H_6_AsO_2_Na (pH 5) | C1 | C2 | C3 | C4 | C5 | C6 | 4000 |
| C_2_H_6_AsO_2_Na (pH 5.5) | D1 | D2 | D3 | D4 | D5 | D6 | 4000 |
| C_2_H_6_AsO_2_Na (pH 6) | E1 | E2 | E3 | E4 | E5 | E6 | 4000 |

All conditions contained 0.2 M ([N](http://en.wikipedia.org/wiki/Nitrogen)[H](http://en.wikipedia.org/wiki/Hydrogen)_4_)_2_[S](http://en.wikipedia.org/wiki/Sulfur)[O](http://en.wikipedia.org/wiki/Oxygen)_4_

**Table 2A: TOPS2 screen conditions 1-48**

| Buffer (pH) (0.1 M) or non buffered component (0.2 M) | PEG 4000 | | | | | |
| --- | --- | --- | --- | --- | --- | --- |
|  | 10% | 12.5% | 15% | 17.5% | 20% | 22.5% |
| Na_2_SO_4_ | A1 | A2 | A3 | A4 | A5 | A6 |
| C_2_H_6_AsO_2_Na (pH 6.5) | B1 | B2 | B3 | B4 | B5 | B6 |
| MES (pH 7) | C1 | C2 | C3 | C4 | C5 | C6 |
| HEPES (pH 7) | D1 | D2 | D3 | D4 | D5 | D6 |
| HEPES (pH 7.5) | E1 | E2 | E3 | E4 | E5 | E6 |
| TRIS (pH 7.5) | F1 | F2 | F3 | F4 | F5 | F6 |
| TRIS (pH 8) | G1 | G2 | G3 | G4 | G5 | G6 |
| TRIS (pH 8.5) | H1 | H2 | H3 | H4 | H5 | H6 |

All conditions contained 0.2 M ([N](http://en.wikipedia.org/wiki/Nitrogen)[H](http://en.wikipedia.org/wiki/Hydrogen)_4_)_2_[S](http://en.wikipedia.org/wiki/Sulfur)[O](http://en.wikipedia.org/wiki/Oxygen)_4_

**Table 2B: TOPS2 screen conditions 49-96**

| Buffer (pH) (0.1 M) or non buffered component (0.2 M) | PEG 8000 | | | | | |
| --- | --- | --- | --- | --- | --- | --- |
|  | 10% | 12.5% | 15% | 17.5% | 20% | 22.5% |
| Na_2_SO_4_ | A7 | A8 | A9 | A10 | A11 | A12 |
| C_2_H_6_AsO_2_Na (pH 6.5) | B7 | B8 | B9 | B10 | B11 | B12 |
| MES (pH 7) | C7 | C8 | C9 | C10 | C11 | C12 |
| HEPES (pH 7) | D7 | D8 | D9 | D10 | D11 | D12 |
| HEPES (pH 7.5) | E7 | E8 | E9 | E10 | E11 | E12 |
| TRIS (pH 7.5) | F7 | F8 | F9 | F10 | F11 | F12 |
| TRIS (pH 8) | G7 | G8 | G9 | G10 | G11 | G12 |
| TRIS (pH 8.5) | H7 | H8 | H9 | H10 | H11 | H12 |

All conditions contained 0.2 M ([N](http://en.wikipedia.org/wiki/Nitrogen)[H](http://en.wikipedia.org/wiki/Hydrogen)_4_)_2_[S](http://en.wikipedia.org/wiki/Sulfur)[O](http://en.wikipedia.org/wiki/Oxygen)_4_

**Table 3A: TOPS3 screen conditions 1-48**

| Buffer (pH) (0.1 M) | PEG 4000 | | | | | |
| --- | --- | --- | --- | --- | --- | --- |
|  | 15% | 20% | 25% | 15% | 20% | 25% |
| C_2_H_6_AsO_2_Na (pH 6.5) | A1 | A2 | A3 | A4 | A5 | A6 |
| MES (pH 6.5) | B1 | B2 | B3 | B4 | B5 | B6 |
| MES (pH 7) | C1 | C2 | C3 | C4 | C5 | C6 |
| HEPES (pH 7) | D1 | D2 | D3 | D4 | D5 | D6 |
| TRIS Propane (pH 7) | E1 | E2 | E3 | E4 | E5 | E6 |
| TRIS (pH 7) | F1 | F2 | F3 | F4 | F5 | F6 |
| TRIS (pH 7.5) | G1 | G2 | G3 | G4 | G5 | G6 |
| HEPES (pH 7.5) | H1 | H2 | H3 | H4 | H5 | H6 |
|  | 0% | 0% | 0% | 4.4% | 4.4% | 4.4% |
|  | Glycerol | | | | | |

**Table 3B: TOPS3 screen conditions 49-96**

| Buffer (pH) (0.1 M) | PEG 4000 | | | | | |
| --- | --- | --- | --- | --- | --- | --- |
|  | 15% | 20% | 25% | 15% | 20% | 25% |
| C_2_H_6_AsO_2_Na (pH 6.5) | A7 | A8 | A9 | A10 | A11 | A12 |
| MES (pH 6.5) | B7 | B8 | B9 | B10 | B11 | B12 |
| MES (pH 7) | C7 | C8 | C9 | C10 | C11 | C12 |
| HEPES (pH 7) | D7 | D8 | D9 | D10 | D11 | D12 |
| TRIS Propane (pH 7) | E7 | E8 | E9 | E10 | E11 | E12 |
| TRIS (pH 7) | F7 | F8 | F9 | F10 | F11 | F12 |
| TRIS (pH 7.5) | G7 | G8 | G9 | G10 | G11 | G12 |
| HEPES (pH 7.5) | H7 | H8 | H9 | H10 | H11 | H12 |
|  | 8.7% | 8.7% | 8.7% | 17.4% | 17.4% | 17.4% |
|  | Glycerol | | | | | |

**Table 4A: TOPS4 screen conditions 1-48**

| Buffer (pH) (0.1 M) | PEG 4000 | | | | | |
| --- | --- | --- | --- | --- | --- | --- |
|  | 15% | 20% | 25% | 15% | 20% | 25% |
| C_2_H_6_AsO_2_Na (pH 6.5) | A1 | A2 | A3 | A4 | A5 | A6 |
| MES (pH 6.5) | B1 | B2 | B3 | B4 | B5 | B6 |
| MES (pH 7) | C1 | C2 | C3 | C4 | C5 | C6 |
| HEPES (pH 7) | D1 | D2 | D3 | D4 | D5 | D6 |
| TRIS Propane (pH 7) | E1 | E2 | E3 | E4 | E5 | E6 |
| TRIS (pH 7) | F1 | F2 | F3 | F4 | F5 | F6 |
| TRIS (pH 7.5) | G1 | G2 | G3 | G4 | G5 | G6 |
| HEPES (pH 7.5) | H1 | H2 | H3 | H4 | H5 | H6 |
|  | 0% | 0% | 0% | 4.4% | 4.4% | 4.4% |
|  | Glycerol | | | | | |

All conditions contained 0.2 M ([N](http://en.wikipedia.org/wiki/Nitrogen)[H](http://en.wikipedia.org/wiki/Hydrogen)_4_)_2_[S](http://en.wikipedia.org/wiki/Sulfur)[O](http://en.wikipedia.org/wiki/Oxygen)_4_

**Table 4B: TOPS4 screen conditions 49-96**

| Buffer (pH) (0.1 M) | PEG 4000 | | | | | |
| --- | --- | --- | --- | --- | --- | --- |
|  | 15% | 20% | 25% | 15% | 20% | 25% |
| C_2_H_6_AsO_2_Na (pH 6.5) | A7 | A8 | A9 | A10 | A11 | A12 |
| MES (pH 6.5) | B7 | B8 | B9 | B10 | B11 | B12 |
| MES (pH 7) | C7 | C8 | C9 | C10 | C11 | C12 |
| HEPES (pH 7) | D7 | D8 | D9 | D10 | D11 | D12 |
| TRIS Propane (pH 7) | E7 | E8 | E9 | E10 | E11 | E12 |
| TRIS (pH 7) | F7 | F8 | F9 | F10 | F11 | F12 |
| TRIS (pH 7.5) | G7 | G8 | G9 | G10 | G11 | G12 |
| HEPES (pH 7.5) | H7 | H8 | H9 | H10 | H11 | H12 |
|  | 8.7% | 8.7% | 8.7% | 17.4% | 17.4% | 17.4% |
|  | Glycerol | | | | | |

All conditions contained 0.2 M ([N](http://en.wikipedia.org/wiki/Nitrogen)[H](http://en.wikipedia.org/wiki/Hydrogen)_4_)_2_[S](http://en.wikipedia.org/wiki/Sulfur)[O](http://en.wikipedia.org/wiki/Oxygen)_4_
